# Testing breast cancer serum biomarkers for early detection and prognosis in pre-diagnosis samples

**DOI:** 10.1038/bjc.2016.433

**Published:** 2017-01-12

**Authors:** Anna Kazarian, Oleg Blyuss, Gergana Metodieva, Aleksandra Gentry-Maharaj, Andy Ryan, Elena M Kiseleva, Olga M Prytomanova, Ian J Jacobs, Martin Widschwendter, Usha Menon, John F Timms

**Affiliations:** 1 Department of Women's Cancer, Institute for Women's Health, University College London, London, W1T 7DN, UK; 2 Department of Biological Sciences, University of Essex, Colchester, Essex CO4 3SQ, UK; 3 Oles Honchar Dnipropetrovsk National University, Dnipropetrovsk, Ukraine; 4 University of New South Wales, Sydney, NSW 2052, Australia

**Keywords:** breast cancer, biomarkers, pre-diagnosis serum, UKCTOCS, CA15-3, PAI-1, HSP90A

## Abstract

**Background:**

Breast cancer is a leading cause of morbidity and mortality worldwide. Although mammography screening is available, there is an ongoing interest in improved early detection and prognosis. Herein, we have analysed a combination of serological biomarkers in a case–control cohort of sera taken before diagnosis.

**Methods:**

This nested case–control study within the UK Collaborative Trial of Ovarian Cancer Screening (UKCTOCS) used serum samples from 239 women who subsequently developed breast cancer and 239 matched cancer-free controls. Sera were screened by ELISA for 9 candidate markers. Univariate and multivariate analyses were performed to examine associations with clinico-pathological features and between case controls in different time groups before diagnosis.

**Results:**

Significant associations with clinico-pathological features related to prognosis were found for several candidates (CA15-3, HSP90A and PAI-1). However, there were no consistent differences between cases and controls for any candidate in the lead up to diagnosis. Whilst combination models outperformed single markers, there was no increase in performance towards diagnosis.

**Conclusions:**

This study using unique pre-diagnosis samples shows that CA15-3, HSP90A and PAI-1 have potential as early prognostic markers and warrant further investigation. However, none of the candidates or combinations would be useful for screening.

Breast cancer is the most common neoplasm in women and the second leading cause of cancer-related mortality in females worldwide (Siegel *et al*, 2014). At present, breast cancer detection relies mostly on mammography, which has been associated with decreased breast cancer mortality (Jatoi, 1999; Gotzsche and Jorgensen, 2013). However, mammography screening has generated controversy due to the risks of false-positive results and over-diagnosis of indolent disease (Baum, 2010; Gotzsche and Jorgensen, 2013; Pace and Keating, 2014; Welch *et al*, 2016). Mammography also has limited sensitivity for the detection of tumours in dense breast tissue (Boyd *et al*, 2007). There is thus an urgent need for early biomarkers that could predict disease outcome, providing prognostic information to the clinician for treatment stratification. The addition of a blood-based tumour marker test may also increase patient compliance as blood testing is more acceptable and would also circumvent the problems associated with imaging high-density breast tissue.

Some blood-borne tumour markers have demonstrated ability to detect malignancy before clinical diagnosis and are currently being evaluated in screening trials for certain cancers; for example, CA125 for screening ovarian cancer (Menon *et al*, 2015). Breast cancer markers in clinical practice are used for predicting response to therapy, monitoring after primary therapy or as prognostic indicators (Harris *et al*, 2007). However, there are currently no blood-borne biomarkers recommended for breast cancer diagnosis or screening. Although candidate markers such as carcinoembryonic antigen (CEA), the soluble form of MUC1 protein (CA15-3, CA27.29) and circulating cytokeratin fragments (TPA, TPS and CYFRA 21-1) have been suggested as diagnostic markers, they lack sensitivity for early disease detection and/or lack specificity.

Almost all diagnostic biomarker studies to date have used samples collected at or just after diagnosis and therefore may be confounded by late-stage responses to advanced tumours. Ideally, samples taken before clinical diagnosis should be used in the search for biomarkers of pre-symptomatic, early stage disease and for predicting prognosis. Herein, we have sourced such pre-diagnostic samples from the UK Collaborative Trial of Ovarian Cancer Screening (UKCTOCS); a multi-centre RCT (the largest ever undertaken), which aims to assess if screening for ovarian cancer can save lives (Menon *et al*, 2009, 2015). Serum samples were taken from 239 UKCTOCS women who were diagnosed with invasive ductal carcinoma of the breast, months to years after sample donation. Cases were matched 1 : 1 with samples from non-cancer controls. The serum biomarkers investigated in this study were chosen based on previous evidence of their potential as serum and/or tissue markers of breast cancer: CA15-3 (cancer antigen 15-3) (Hayes *et al*, 1986; Geraghty *et al*, 1992; Stieber *et al*, 2003), RANTES/CCL5 (regulated on activation, normal T cell expressed and secreted/chemokine (C–C motif) ligand 5) (Soria and Ben-Baruch, 2008; Gonzalez *et al*, 2011), OPN (osteopontin) (Singhal *et al*, 1997; Fedarko *et al*, 2001; Bramwell *et al*, 2014), PAI-1 (plasminogen activator inhibitor-1; Harris *et al*, 2007; Duffy *et al*, 2014), SLPI (secretory leukocyte protease inhibitor) (Hu *et al*, 2004; Bouchard *et al*, 2006), HSP90A (heat shock protein 90A; Pick *et al*, 2007; Duran *et al*, 2008; Cheng *et al*, 2012), IGFBP3 (insulin-like growth factor-binding protein 3; McCarthy *et al*, 2009; Worthington *et al*, 2010), APOC1 (apolipoprotein C-I) (Opstal-van Winden *et al*, 2011; Devetyarov *et al*, 2012; Chung *et al*, 2014; Lee *et al*, 2015) and PAPPA (pappalysin-1; Loddo *et al*, 2014; Mansfield *et al*, 2014). Herein we report on the ability of these serum markers to detect breast cancer cases before their diagnosis and examine associations with clinico-pathological features and prognostic indicators.

## Materials and Methods

### Ethics, consent and permissions

UKCTOCS participants gave informed written consent at recruitment for the use of their medical notes and serum in ethically approved secondary studies (UK North West Medical Research and Ethics Committee (MREC 00/8/34)). Ethical approval for this nested case–control study was granted by The Joint UCL/UCLH Committees on the Ethics of Human Research (Committee A) REC ref 05/Q0505/57.

### Subjects

UKCTOCS participants were post-menopausal women aged 50–74, who had no active malignancy at recruitment (Menon *et al*, 2008). Notifications of women subsequently diagnosed with invasive ductal carcinoma of the breast were retrieved by querying the Health and Social Care Information Centre (HSCIC) cancer and death registries with the International Classification of Diseases code C50 pertaining to malignant neoplasms of the breast. Cancer notifications were also received via self-reported data completed 3.5 years post-randomisation to the UKCTOCS. Breast cancer notifications were confirmed and characterised by postal questionnaire sent to treating clinicians (consultant, or General Practitioner if details not provided by the volunteer), which was designed to ascertain clinical and histological data on diagnosed cases (date of diagnosis, histology, nodal status, staging, grade, prognosis, ER, PR and HER2 status). Staging for each case was determined using the TNM system, taking into account tumour size (T), spread to the lymph nodes (N), and whether the tumour had metastasised (M). To provide an indication of prognosis, the Nottingham prognostic index (NPI) was calculated for each case using the formula [0.2 × *S*]+*N*+*G*, where S=tumour size in cm, N=node status (0 nodes=1, 1–4 nodes=2, >4 nodes=3) and G=numerical grade. Cases were stratified into good and poor prognosis using an NPI ⩽4 and >4, respectively. Controls were age-matched and were women from the same trial centre who had no history of cancer, and who had donated serum samples on the same date as the matched case. Characteristics of the case–control set are shown in Table 1. There was no significant difference in time to spin (clotting time) or age between cases and controls.

### Samples and assays

Blood was collected and serum prepared according to a standardised protocol within the UKCTOCS (Menon *et al*, 2009) and then shipped frozen to a cryo-repository for long-term storage in liquid nitrogen. For the study, single samples from cases were selected that predated diagnosis by a median time of 1.15 years (13.8 months; range 0.1–53.8 months). Samples were retrieved from storage and shipped to the laboratory on dry ice and thawed at 4 °C for random aliquoting before assay. Serum concentrations of all biomarker candidates were measured using commercial enzyme-linked immunosorbent assays (ELISA) according to the manufacturers' instructions. Optimum serum dilutions were first determined using a pool of serum from cases and controls and used to determine inter-assay CVs. The ELISA kits used, dilutions and inter-assay CVs were; CA15-3 (Human) ELISA kit (Abnova; 1 : 50; 7.3%), Human CCL5/RANTES Quantikine ELISA (R&D; 1 : 100; 8.1%), Human IGFBP-3 Quantikine ELISA (R&D; 1 : 100; 9.8%), Human PAI-1 ELISA Kit (Invitrogen; 1 : 25; 8.1%), Human Osteopontin (OPN) Quantikine ELISA (R&D; 1 : 12.5; 6.1%), Hsp90*α* ELISA Kit (Stressgen; 1 : 30; 6.5%), Human SLPI Quantikine ELISA (R&D; 1 : 30; 8.5%), human Pappalysin-1 (PAPPA) ELISA kit (DRG; 1 : 200; 11.3%) and human Apolipoprotein C-I (APOC1) ELISA kit (Abnova; 1 : 100; 11.1%). There were no strong correlations with clotting time or volunteer age for any of the measured analytes.

### Statistical analysis

Univariate analysis using GraphPad Prism software (v5.01) was used to test associations between analyte measurements and clinico-pathological features and between cases and matched controls in different time groups before diagnosis. For normally distributed data, the Student *t*-test was used to assess significance of differences; otherwise the Mann–Whitney *U*-test was used. *P*-values of <0.05 were considered significant. Kaplan–Meier analysis was used to examine biomarker levels in relation to survival in cases using time from sample collection to death and various candidate marker cut-off values. The log-rank test was used to test significance between the survival curves. Multivariate logistic regression models were constructed and tested within the R environment (v3.1.1 for Mac OS). Evaluation of performance of each model was based on receiver operating characteristic (ROC) curve analysis, with determination of significance of differences in areas under the curves using the method of DeLong *et al* (1988). A modelling approach based on the neuro-fuzzy technique was also applied to the data, which combines the mathematical constructions of fuzzy logic (Nauck *et al*, 1997; Paiva and Dourado, 2004) and neural networks. Briefly, a fuzzy model was generated using half of the data and its parameters estimated to minimise the difference between the model and the experimental data. Model adjustment was based on the sub-gradient modification method for the case of undifferentiated functions, the so-called r-algorithms by Shor (Kiseleva and Shor, 2005), with an accuracy of *ɛ*=0.001. The model was then validated on the other half of the data. Software implementation of the approach was developed within the Visual C++ environment.

## Results

### Pre-diagnostic candidate marker levels in discrimination of case–control samples

In this UKCTOCS nested case–control set, volunteers donated serum up to 5 years before clinical diagnosis of breast cancer, with a median time to diagnosis of 1.15 years (13.8 months). We hypothesised that serum levels of the candidate markers may be altered in women with a clinically undetectable tumour. Initially, biomarker data from all case samples were compared with all non-cancer controls and then those taken less than and greater than the median time to diagnosis. No single candidate marker showed a significant difference between cases and controls for these comparisons. Samples were also stratified into time groups that came from cases who developed breast cancer within 0.5 years, 0.5–1 years, 1–2 years, 2–3 years and 3–5 years of sample collection and candidate marker levels were compared against matched-control samples for these groups. Overall, there were no consistent changes in serum concentrations for any marker in cases in the lead up to diagnosis, although some significant differences between cases and controls were apparent for some candidates in different time groups. Thus, cases had elevated levels of IGFBP3 (*P*=0.044) 0–0.5 years before diagnosis, lower levels of RANTES (*P*=0.013) 2–3 years before diagnosis and higher levels of APOC1 (*P*=0.011) and lower levels of PAPPA (*P*=0.036) at 3–5 years before diagnosis.

Combining markers (including age as a parameter) using logistic regression failed to provide accurate discrimination of cases and controls in any pre-diagnosis time group, although combined models performed better than the single markers for classification (Table 2). There was no trend of improved classification in the lead up to diagnosis and sensitivities were in the range 4–26% at a specificity of >95%. A novel modelling approach based on the neuro-fuzzy technique also failed to provide accurate models, with sensitivities in the range 10–27% (data not shown). These data suggest that these candidates alone or combination are not accurate markers for predicting breast cancer.

### Pre-diagnostic candidate marker levels in relation to clinico-pathological features

**Figure 1:**
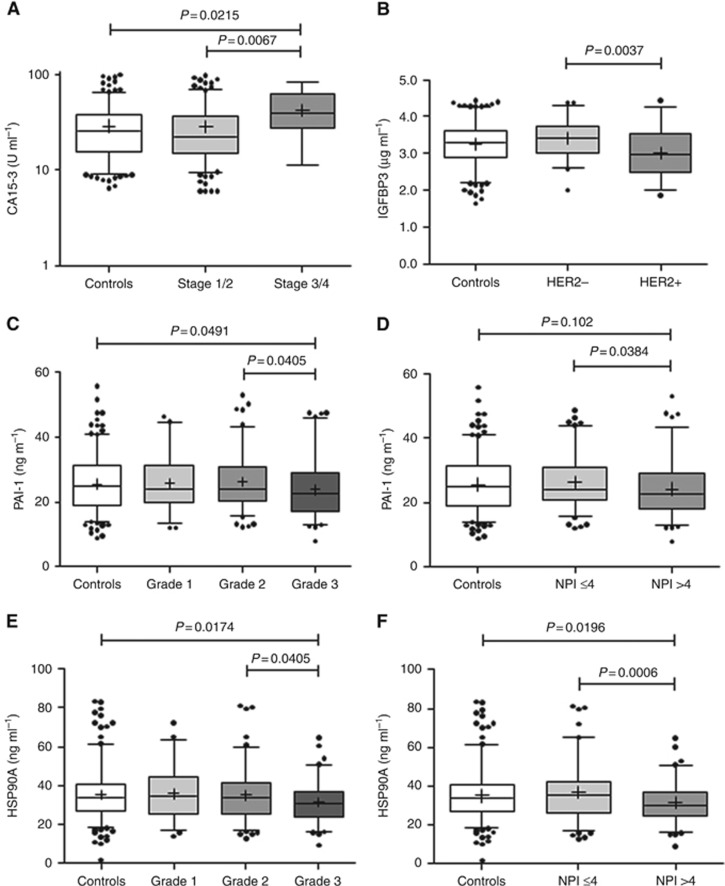
**Associations of biomarkers with histopathological features.** Box and whisker plots showing associations of CA15-3 with tumour stage (**A**); IGFBP3 with HER2 status in the <1.15 years to diagnosis group (**B**); PAI-1 with tumour grade (**C**); PAI-1 with NPI (**D**); HSP90A with tumour grade (**E**); and HSP90A with NPI in the <1.15 years to diagnosis group (**F**).

Pre-diagnostic serum levels of CA15-3 were significantly raised in samples from late-stage cases (stages 3/4 combined) *vs* controls and stage 1 and stage 2 cases (Tables 3A and 4; Figure 1A) and with node positivity within cases (Table 4). The marker was also raised significantly in node positive cancers within cases (Table 4) and *vs* controls in the subset of cases from which samples were taken <1.15 years before clinical diagnosis (Table 3B). However, CA15.3 levels were lower in grade 1 cancers *vs* controls or grade 2 cancers. Neither RANTES or OPN discriminated cases from controls for any of the pathological parameters examined (Table 3), and whereas altered levels of both were associated with tumour stage at diagnosis within cases (Table 4), for RANTES, this was not seen in the <1.15 year to diagnosis group and for OPN, the changes were not consistent, with lower levels in stage 2 *vs* stage 1 or stage 3/4 cases. IGFBP3 also failed to discriminate cases from controls, although was decreased in case samples that went on to be diagnosed with HER2+ breast cancer where there was a strong association for the <1.15 years to diagnosis group (Table 4B; Figure 1B). Serum levels of PAI-1 were significantly lower in samples from women who went on to be diagnosed with grade 3 cancer *vs* controls and grade 2 cancers and within cases with a higher NPI (Figures 1C and D). Similarly, HSP90A levels were lower in pre-diagnosis serum of grade 3 cases *vs* controls and in cases with a higher NPI (Figures 1E and F,) with the differences more significant closer to diagnosis (Tables 3 and 4). The association of lowered HSP90A with prognostic index was also significant (*P*=0.012) when only node-negative cases were considered. SLPI, PAPPA and APOC1 levels did not differ significantly for any of the pathological parameters and there were no differences for any candidate markers in relation to oestrogen receptor, progesterone receptor or triple negative status. The best logistic regression models combining markers for prognosis based on NPI gave AUCs of 0.67 (HSP90A, PAI-1 and RANTES) considering all samples, and 0.77 (HSP90A, PAI-1 and CA15-3), considering samples taken within 1.15 years of diagnosis. Although the AUCs for candidate markers were higher when comparing controls with cases that had subsequently died of breast cancer *vs* those that had not, a survival analysis indicated that none of the markers were predictive of survival from breast cancer (data not shown). However, this analysis is somewhat limited, as there were only 17 of the 239 cases (7%) where the primary cause of death was attributable to breast cancer (median time to death from sample collection=6.12 years; range 2.1–11.1 years).

## Discussion

Few studies have tested blood-borne candidate tumour markers for early detection or prognosis in pre-diagnosis samples; the most appropriate sample type for this kind of study. Here, we found that none of the single marker candidates were effective at predicting breast cancer, even close to diagnosis, and there was lack of consistent changes in levels over time for any of the candidates. Although combining the candidate markers improved the detection rat, no combined model performed with a sensitivity that would be acceptable for use in screening. This is in agreement with a previous study using a smaller set of pre-diagnostic samples sourced from the Prospect-EPIC cohort (Opstal-van Winden *et al*, 2012). The authors reported that no marker combination (of 10 candidates, including CA15-3 and OPN) could accurately discriminate early breast cancer cases from controls. Thus, novel blood-borne biomarkers for the early detection of breast cancer for use in screening still need to be found.

CA15-3 is perhaps the best known, non-invasive marker of breast cancer, although its recommended clinical use is restricted to monitoring of patients with metastatic disease during active therapy (Harris *et al*, 2007). Herein, CA15-3 levels in serum taken at a median time of 13.8 months before clinical diagnosis showed consistency with respect to stage, grade and nodal status within cases, although it failed to discriminate cases from controls. This suggests that CA15-3 may serve as a pre-diagnostic marker of a more aggressive tumour phenotype, in line with previous reports of the prognostic value of CA15-3 in breast cancer (Ebeling *et al*, 2002; Gion *et al*, 2002; Shao *et al*, 2015). Raised pre-diagnostic serum levels of the chemoattractant RANTES were also associated with stage within cases, supporting previous observations of raised plasma and tissue expression correlating with increasing stage (Niwa *et al*, 2001). Notably however, this association was not significant in cases where samples were taken closer to diagnosis, suggesting RANTES is not a robust predictor of stage. Similarly, stage-associated changes in serum OPN levels were not consistent, and this bone adhesion molecule could not differentiate cases by grade, node status or prognostic index. This supports previous evidence that OPN is not a useful prognostic marker in early breast cancer (Bramwell *et al*, 2014).

The fibrinolysis regulator protein PAI-1 has reported roles in invasion, angiogenesis and metastasis with high levels in breast tumour tissue having prognostic value and use in stratification, particularly of node-negative breast cancer (Look *et al*, 2002; Duffy *et al*, 2014). Few studies have assessed blood-borne PAI-1 as a breast tumour marker, with one study reporting decreased serum concentrations in cases *vs* non-cancer controls (Kim *et al*, 2009). Herein, serum PAI-1 was reduced in women who went on to be diagnosed with grade 3 breast cancer or who had a high prognostic index, though it was unable to discriminate pre-diagnosis cases from controls. Although this data supports serum PAI-1 as an early prognostic factor, its direction of change is at odds with the raised levels seen in tumour tissues. HSP90A is a chaperone protein for several oncogenes (including HER2) and a pro-survival factor of breast cancer cells. We showed lower levels of serum HSP90A in women who went on to be diagnosed with high-grade cancers and with higher prognostic index. High tissue expression of HSP90 has been previously associated with increased HER2 and ER expression, large and high-grade tumours, node positivity and decreased survival (Pick *et al*, 2007), although another study reported no significant association between serum HSP90A levels and lesion severity (Zagouri *et al*, 2011). These combined evidences suggest that serum HSP90A may not make a good early prognostic marker, although based on our findings, further investigation of its potential is warranted. Finally, the serum level of the IGF1-binding protein IGFBP3 was significantly lower in samples from HER2+ cases taken within 1.15 years of diagnosis. This observation is in accordance with our previous finding that HER2 overexpression enhances IGF1 signalling via the down-regulation of IGFBP3 (Worthington *et al*, 2010). However, although increased serum IGF1 levels have been associated with increased breast cancer risk, no association with serum IGFBP3 levels was found (Key *et al*, 2010). Thus, IGFBP3 is a poor marker for predicting breast cancer, although the relationship between HER2 and IGFBP3 expression warrants further investigation.

## Conclusions

The key strength of this study is that a well-characterised, pre-diagnosis set of samples were investigated, allowing an objective assessment of nine candidate serological markers for the early detection and prognostication of breast cancer. One weakness of the study is that more detailed information on the molecular sub-type of the tumours was not available (excepting receptor status), so future studies should address in more detail the possible effect of molecular sub-type on candidate biomarker levels, as recently reported for CA15-3 (Shao *et al*, 2015). We conclude that CA15-3, PAI-1 and HSP90A have potential as prognostic markers in the pre-diagnosis setting. The next phase of this work would be to validate these candidate biomarkers in a larger independent set of case–control samples sourced from the UKCTOCS with data linked to mammography findings, treatment and survival information for these women. Serial samples from breast cancer cases and matched controls would be used from the multimodal arm of the UKCTOCS, permitting assessment of longitudinal changes in these candidate biomarkers during tumour progression. We envisage, that if validated, these markers could be used as a second line test to mammography to identify tumours of poorer prognosis and thereby allow stratification for treatment. Whether or not such testing would have an impact on survival would need to be assessed in a prospective clinical trial. We also conclude that CA15-3, RANTES, IGFBP3, OPN, PAI-1, SLPI, HSP90A, PAPPA and APOC1 alone, or in combination, cannot be used for accurate prediction of breast cancer and therefore would be of no use in screening. Despite this, our work lays the groundwork for building and assessing longitudinal biomarker algorithms that may give an improvement in performance, such as the Risk of Ovarian Cancer Algorithm used within the UKCTOCS (Menon *et al*, 2015).

## Figures and Tables

**Table 1:** Study set and clinico-pathological features

**Variable**	**Cases**	**Controls**	***P*****-value**
No. individuals	239	239	
No. samples	239	239	
Mean time to spin (h; range)	24.05 (1.83–47.69)	23.87 (1.77–47.78)	0.52
Mean age at sample draw (years; range)	61.24 (50.6–75.9)	60.78 (50.3–76.5)	0.3
Median time from sample draw to diagnosis (months; range)	13.84 (0.13–53.81)		
Tumour grade (number of cases)			
1	47		
2	111		
3	81		
Node status (number of cases)			
N0	176		
N1	63		
Stage (number of cases)			
1	115		
2	87		
3	12		
4	2		
Not assessed	23		
Metastasis (number of cases)			
M0	166		
M1	2		
Not assessed (Mx)	28		
Not available	43		
NPI estimate (number of cases)			
⩽4.0	107		
>4.0	96		
Not available	36		
HER2 status (number of cases)			
Negative	136		
Positive	47		
Not available	56		
ER status (number of cases)			
Negative	47		
Positive	191		
Unknown	1		
PR status (number of cases)			
Negative	84		
Positive	132		
Not available	23		

Abbreviation: NPI=Nottingham prognostic index.

**Table 2:** Performance of logistic regression models of single and multiple candidate biomarkers according to time to diagnosis

**Time group (years)**	**Best single marker**	**AUC of best marker**	**Best combination (powers for 9 parameters, i1:i9)**	**AUC of best combination**	***P*****-value for AUC**	**Sens for best combination (Spec>0.95)**
0–5 (All)	HSP90A	0.529	(−2; 2; −2; −2; 0; 1; −2; −2; −2)	0.56	0.224	0.096
0–1.15	SLPI	0.533	(1; −1; 2; −2; 0; 2; −2; 1; 2)	0.573	0.203	0.083
1.15–5	PAPPA	0.559	(1; 1; −1; −1; 2; 1; 1; 1; −1)	0.593	0.208	0.042
0–0.5	IGFBP3	0.605	(2; 1; −2; −2; 2; −2; 2; 2; 2)	0.686	0.047	0.179
0.5–1	APOC1	0.601	(0; 1; 2; −1; 1; −1; −2; −2; 2)	0.752	0.007	0.189
1–2	RANTES	0.572	(−2; 2; −2; 2; 2; −1; −2; −1; 2)	0.675	0.08	0.14
2–3	RANTES	0.669	(−2; 2; −1; 2; 1; 2; −2; −2; 1)	0.781	0.048	0.257
3–5	APOC1	0.685	(−1; −2; −2; −2; 1; 1; 1; 2; −2)	0.785	0.06	0.216

Performances are indicated by area under the ROC curve (AUC). For the best combinations, powers are indicated for the formula: (IGFBP3)^i1^+(RANTES)^i2^+(OPN)^i3^+(PAI-1)^i4^+(SLPI)^i5^+(HSP90A)^i6^+(APOC1)^i7^+(PAPPA)^i8^+(CA15-3)^i9^+(Age).

**Table 3:** Comparison of cases and controls for candidate biomarkers according to stage, grade, node status, NPI and HER2 status

	**Stage**		**Grade**			**Node status**		**NPI**		**HER2 status**	
**Marker**	**Ctrl** ***vs*** **1/2**	**Ctrl** ***vs*** **3/4**	**Ctrl** ***vs*** **1**	**Ctrl** ***vs*** **2**	**Ctrl** ***vs*** **3**	**Ctrl** ***vs*** **N0**	**Ctrl** ***vs*** **N1**	**Ctrl** ***vs*** **⩽4**	**Ctrl** ***vs*** **>4**	**Ctrl** ***vs*** **neg**	**Ctrl** ***vs*** **pos**
**(A) All samples**											
CA15-3	NS	0.0215	0.0254	NS	NS	NS	NS	NS	NS	NS	NS
RANTES	NS	NS	NS	NS	NS	NS	NS	NS	NS	NS	NS
OPN	NS	NS	NS	NS	NS	NS	NS	NS	NS	NS	NS
IGFBP3	NS	NS	NS	NS	NS	NS	NS	NS	NS	NS	NS
PAI-1	NS	NS	NS	NS	0.0491	NS	NS	NS	NS	NS	NS
HSP90A	NS	NS	NS	NS	0.0174	NS	NS	NS	0.0196	ns	ns
SLPI	NS	NS	NS	NS	NS	NS	NS	NS	NS	NS	NS
PAPPA	NS	NS	NS	NS	NS	NS	NS	NS	NS	NS	NS
APOC1	NS	NS	NS	NS	NS	NS	NS	NS	NS	NS	NS
**(B) <1.15 years to D*****x***											
CA15-3	NS	NS	NS	NS	NS	NS	0.0419	NS	NS	NS	NS
RANTES	NS	NS	NS	NS	NS	NS	NS	NS	NS	NS	NS
OPN	NS	NS	NS	NS	NS	NS	NS	NS	NS	NS	NS
IGFBP3	NS	NS	NS	NS	NS	NS	NS	NS	NS	NS	NS
PAI-1	NS	NS	NS	NS	NS	NS	NS	NS	NS	NS	NS
HSP90A	NS	NS	NS	NS	NS	NS	NS	0.0259	0.0419	NS	NS
SLPI	NS	NS	NS	NS	NS	NS	NS	NS	NS	NS	NS
PAPPA	NS	NS	NS	NS	NS	NS	NS	NS	NS	NS	NS
APOC1	NS	NS	NS	NS	NS	NS	NS	NS	NS	NS	NS
**(C) >1.15 years to D*****x***											
CA15-3	NS	NS	0.039	NS	NS	NS	NS	NS	NS	NS	NS
RANTES	NS	NS	NS	NS	NS	NS	NS	NS	NS	NS	NS
OPN	NS	NS	NS	NS	NS	NS	NS	NS	NS	NS	NS
IGFBP3	NS	NS	NS	NS	NS	NS	0.0447	NS	NS	NS	NS
PAI-1	NS	NS	NS	NS	NS	NS	NS	NS	NS	0.0391	0.0364
HSP90A	NS	NS	NS	NS	0.0079	NS	NS	NS	NS	NS	NS
SLPI	NS	NS	NS	NS	NS	NS	NS	NS	NS	NS	NS
PAPPA	NS	NS	NS	NS	NS	NS	NS	NS	NS	NS	NS
APOC1	NS	NS	NS	NS	NS	NS	NS	NS	NS	NS	NS

*P*-values for various comparisons are shown. *P*<0.05 was considered significant; NS denotes non-significance. Comparisons are shown for all pre-diagnosis case samples *vs* all controls (A), between case samples taken <1.15 years to diagnosis (median) and all controls (B) and between case samples taken >1.15 years to diagnosis and all controls (C).

**Table 4:** Analysis of candidate biomarkers within cases according to stage, grade, node status, NPI and HER2 status

	**Stage**			**Grade**			**Node status**	**NPI**	**HER2 status**
**Marker**	**1** ***vs*** **2**	**1** ***vs*** **3/4**	**2** ***vs*** **3/4**	**1** ***vs*** **2**	**1** ***vs*** **3**	**2** ***vs*** **3**	**N1** ***vs*** **N0**	**⩽4** ***vs*** **>4**	**Pos** ***vs*** **neg**
**(A) All samples**									
CA15-3	NS	0.0001	0.0024	0.0078	NS	NS	0.036	NS	NS
RANTES	0.0232	0.0183	NS	NS	NS	NS	NS	NS	NS
OPN	NS	NS	0.0381	NS	NS	NS	NS	NS	NS
IGFBP3	NS	NS	NS	NS	NS	NS	NS	NS	NS
PAI-1	NS	NS	NS	NS	NS	0.0405	NS	0.0384	NS
HSP90A	NS	NS	NS	NS	NS	NS	NS	0.0062	NS
SLPI	NS	NS	NS	NS	NS	NS	NS	NS	NS
PAPPA	NS	NS	NS	NS	NS	NS	NS	NS	NS
APOC1	NS	NS	NS	NS	NS	NS	NS	NS	NS
**(B) <1.15 years to D*****x***									
CA15-3	NS	0.0049	0.032	NS	NS	NS	0.0368	NS	NS
RANTES	NS	NS	NS	NS	NS	NS	NS	NS	NS
OPN	0.0245	NS	0.0052	NS	NS	NS	NS	NS	NS
IGFBP3	NS	NS	NS	NS	NS	NS	NS	NS	0.0037
PAI-1	NS	NS	NS	NS	NS	NS	NS	0.0331	NS
HSP90A	NS	NS	NS	NS	0.0073	NS	NS	0.0003	NS
SLPI	NS	NS	NS	NS	NS	NS	NS	NS	NS
PAPPA	NS	NS	NS	NS	NS	NS	NS	NS	NS
APOC1	NS	NS	NS	NS	NS	NS	NS	NS	NS

*P*-values are shown for comparisons within all pre-diagnosis case samples for the pathological features (A) and within case samples taken <1.15 years to diagnosis (B). *P*<0.05 was considered significant; NS denotes non-significance.
